# Development and internal validation of the LATE-PE model for predicting time-to-delivery in early-onset preeclampsia: a multicenter study

**DOI:** 10.3389/fmed.2026.1870604

**Published:** 2026-08-28

**Authors:** Van-Du Vu, Quang-Chien Chu, Phuong-Tu Nguyen, Thanh-Tung Do, Van-Tot Dang, Van-Anh Nguyen, Trong-Hung Mai, Thi-Hang Nguyen, Van-Sinh Dang

**Affiliations:** 1 National Hospital of Obstetrics and Gynecology, Hanoi, Vietnam; 2 Hanoi Medical University, Hanoi, Vietnam; 3 Department of Obstetrics and Gynecology, Bach Mai Hospital, Hanoi, Vietnam; 4 Hanoi Obstetrics and Gynecology Hospital, Hanoi, Vietnam

**Keywords:** decision support, nomogram, prediction model, preeclampsia, time-to-delivery

## Abstract

**Introduction:**

Managing early-onset preeclampsia requires balancing maternal risks against neonatal benefits. However, few simple and readily available tools are available to estimate pregnancy latency, particularly in resource-limited settings.

**Methods:**

This multicenter retrospective cohort study developed and internally validated the LATE-PE (Latency Assessment Tool for Early-onset Preeclampsia) model in 266 women with early-onset preeclampsia who had already been considered eligible for expectant management. The model estimates the probability of allcause delivery by day 2 after admission, used as an operational approximation of the clinically relevant 48-hour window, and by day 7. Six routinely available predictors were prespecified: gestational age, systolic blood pressure, proteinuria, AST, creatinine, and platelet count. Internal validation was performed using 1,000 bootstrap resamples.

**Results:**

The apparent AUC was 0.740 (95% CI 0.672–0.808) for delivery by day 2 and 0.767 (95% CI 0.708–0.826) for delivery by day 7, with corresponding optimism-corrected AUCs of 0.732 and 0.758. The optimismcorrected calibration slopes were 1.023 and 0.806 at day 2 and day 7, respectively, and the optimism-corrected C-index was 0.689. Exploratory decision-curve analysis suggested potential net benefit across selected threshold probabilities. An experimental web-based research prototype was developed to facilitate model exploration and future validation.

**Conclusion:**

LATE-PE provides a parsimonious approach to estimating short-term delivery probability using routinely available clinical and laboratory variables. Its accessibility may be particularly relevant where specialized biomarkers or imaging are not readily available. However, the model has undergone internal validation only. External validation in independent populations and prospective assessment of clinical impact are required before clinical use.

## 1 Introduction

Preeclampsia is a complex pregnancy disorder and one of the leading causes of maternal and neonatal morbidity and mortality worldwide, accounting for an estimated 70,000 maternal deaths and approximately 500,000 fetal deaths annually (1, 2). The burden is disproportionately greater in low- and middle-income countries, where preeclampsia and eclampsia contribute to approximately 16% of all maternal deaths (3). This disparity likely reflects socio-economic inequalities and health system limitations that hinder the implementation of modern screening strategies and restrict access to timely diagnosis and appropriate obstetric care. Beyond its immediate obstetric consequences, preeclampsia is associated with substantial short- and long-term health risks for both mothers and their offspring. Offspring exposed to preeclampsia *in utero* may have an increased risk of hypertension, stroke, and cardiovascular diseases later in life (4–6).

Based on the time of onset, preeclampsia is classified into two main types using a threshold of 34 weeks of gestation: early-onset and late-onset (7). Early-onset preeclampsia is generally considered the more severe form, with its pathogenesis closely linked to placental dysfunction. Defective remodeling of the spiral arteries during early pregnancy leads to reduced placental perfusion, triggering hypoxia, oxidative stress, and systemic endothelial dysfunction, thereby increasing the risk of severe complications for both the mother and the fetus (4).

The management of early-onset preeclampsia remains a significant challenge in clinical practice, requiring a careful balance between maternal risks and fetal benefits. Early delivery can reduce the risk of severe maternal complications but is associated with an increased risk of morbidity and mortality related to prematurity. Conversely, expectant management (prolonging the pregnancy) to improve perinatal outcomes may increase the risk of severe maternal and fetal complications (8, 9). In this context, delaying delivery for at least 48 h to complete a course of antenatal corticosteroids, and ideally for more than 7 days, is considered important for improving neonatal outcomes (8–12).

Although these clinical timeframes are widely recognized, decision-making in current practice remains largely dependent on clinician judgment. Existing predictive models predominantly focus on early screening for preeclampsia risk or predicting adverse complications (13–16), whereas prognostic tools estimating the latency period in patients already diagnosed with preeclampsia remain relatively limited (17, 18). Some recent models, such as that developed by Villalaín et al., have taken initial steps to address this issue; however, their requirement for a large number of input variables (up to 15–18 variables), along with specialized tests such as the sFlt-1/PlGF ratio or Doppler ultrasound, may limit their feasibility in routine clinical settings, particularly in resource-limited environments (19).

The primary aim of this study was to develop and internally validate the LATE-PE (Latency Assessment Tool for Early-onset Preeclampsia) model using multicenter data to estimate the probability of all-cause delivery by day 2 and by day 7 after hospital admission in women with early-onset preeclampsia. The model was based on routinely available clinical and laboratory variables measured at the initial assessment, and potential nonlinearity in systolic blood pressure was examined in an exploratory sensitivity analysis. A secondary aim was to develop an experimental web-based research prototype to facilitate model exploration and future validation. The resulting estimates are intended to complement, rather than replace, comprehensive maternal and fetal assessment when considering pregnancy prolongation.

## 2 Materials and methods

### 2.1 Study population

A multicenter retrospective cohort study was conducted among pregnant women diagnosed with early-onset preeclampsia at three tertiary care hospitals: Bach Mai Hospital (2023–2024, Center 1), Bac Ninh Obstetrics and Pediatrics Hospital (2022–2023, Center 2), and Hanoi Obstetrics and Gynecology Hospital (2021–2023, Center 3).

The study population comprised women with singleton pregnancies diagnosed with preeclampsia according to American College of Obstetricians and Gynecologists (ACOG) criteria (20), with early onset defined as diagnosis before 34 + 0 weeks of gestation, who were considered eligible for expectant management at admission.

**Figure 1:**
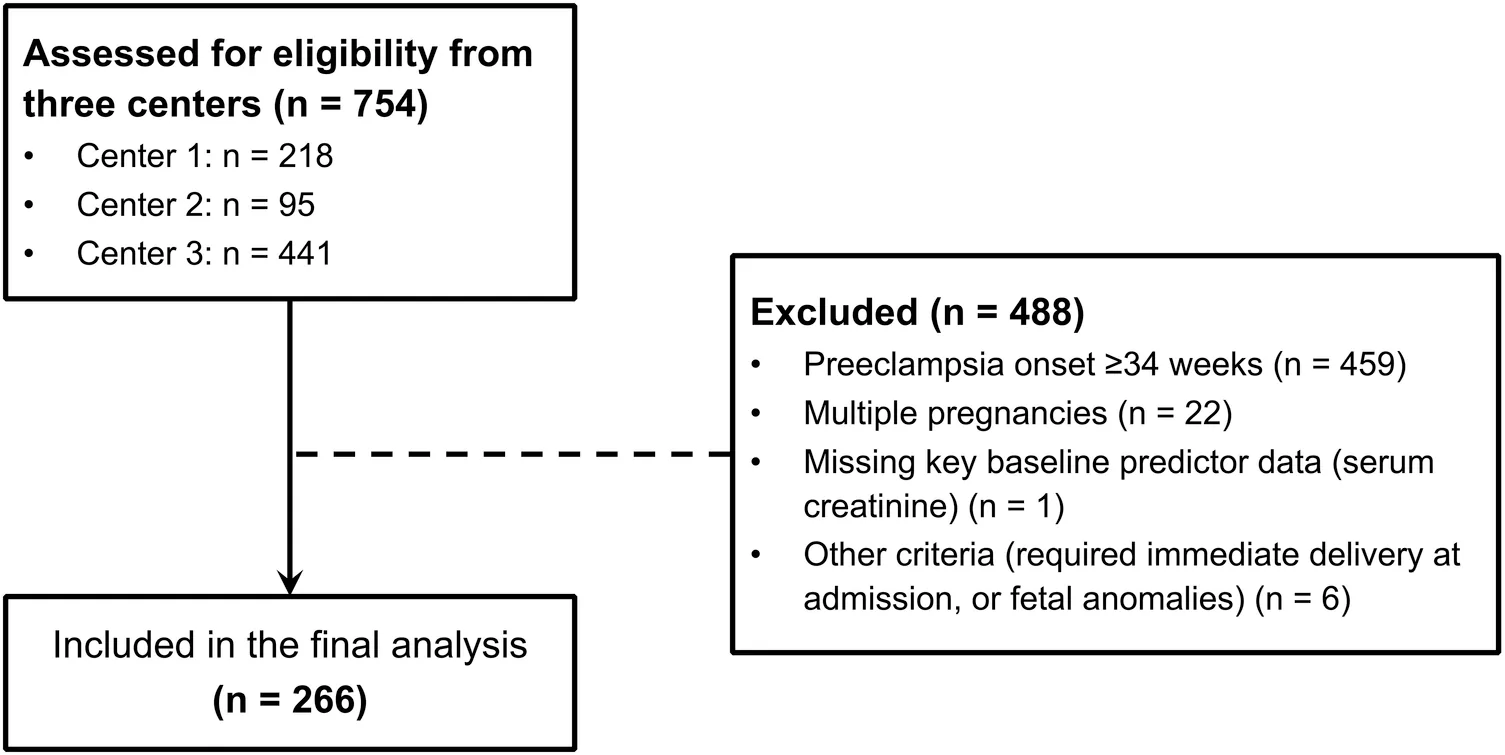
Flowchart of the study population selection process.

Exclusion criteria included: (1) women requiring immediate delivery at admission due to life-threatening maternal or fetal conditions, such as eclampsia, acute pulmonary edema, placental abruption, or non-reassuring fetal status; (2) multiple pregnancies or fetuses with major structural abnormalities, congenital anomalies, or chromosomal abnormalities; and (3) medical records missing any of the six prespecified baseline predictors required for model development or lacking complete follow-up data until delivery. The detailed patient selection process is illustrated in Figure 1.

### 2.2 Study design and sample size

This study was reported in accordance with the TRIPOD + AI guideline for prediction-model studies (21). This retrospective cohort study included participants using a consecutive sampling approach, whereby all eligible cases during the study period were enrolled. All eligible consecutive cases during the study periods were included; therefore, the sample size was determined by the available cohort rather than by an *a priori* calculation. Model complexity was limited *a priori* to six predictor parameters. With 266 delivery events, the final cohort provided approximately 44 events per parameter (22).

### 2.3 Study variables and definitions

The LATE-PE model was developed with an emphasis on parsimony and clinical applicability and comprised six predictors defined at hospital admission. Predictors were selected *a priori* based on clinical relevance, routine availability, and their ability to reflect gestational age, blood-pressure severity, proteinuria, hepatic and renal dysfunction, and thrombocytopenia (7, 20), rather than by significance-based variable selection.

Gestational age was established from first-trimester ultrasonography and expressed as completed weeks plus additional days divided by seven. Systolic blood pressure (measured in mmHg) was the first measurement recorded at admission, before antihypertensive treatment administered at the study hospital. AST (U/L), serum creatinine (*μ*mol/L), platelet count (G/L), and spot urinary protein concentration (g/L) were represented by the result closest to admission during the initial assessment. Proteinuria specifically denoted protein concentration in a spot urine specimen (g/L), not a protein-to-creatinine ratio, 24-hour urinary protein excretion, or dipstick category. The centers did not use identical laboratory analyzers, but results were reported in harmonized units and the laboratories followed national internal and external quality-control requirements.

The primary outcome was time to all-cause delivery. Time zero was hospital admission for the index episode of early-onset preeclampsia. Latency was available at calendar-day resolution and was recorded as the integer number of days from admission to delivery; delivery on the admission date was coded as 0 days. Exact admission and delivery times were unavailable. Therefore, delivery by day 2 (latency ≤2 days) was used as an operational approximation of the 48-hour clinical window, and delivery by day 7 was defined as latency ≤7 days. The indication for delivery could not be classified consistently across centers as maternal, fetal, spontaneous, or combined. All participants were followed until delivery, and no censoring occurred.

### 2.4 Clinical management protocol

Clinical management generally followed institutional protocols aligned with current international guidelines, including ACOG (2020) and ISSHP (2018) (20, 23). Patients were typically admitted for inpatient care to allow close monitoring of maternal and fetal conditions, along with routine laboratory assessments (e.g., complete blood count, platelet count, liver enzymes, and serum creatinine).

Management included blood pressure control using appropriate antihypertensive agents (such as labetalol, hydralazine, or nifedipine), administration of antenatal corticosteroids (betamethasone or dexamethasone) for fetal lung maturation in pregnancies with gestational age <34 + 0 weeks, and magnesium sulfate for seizure prophylaxis and, when indicated, fetal neuroprotection.

Expectant management was terminated and delivery was indicated upon the development of predefined clinical criteria, in accordance with ACOG recommendations (20). Maternal indications included uncontrolled severe hypertension, HELLP syndrome, new-onset or worsening renal dysfunction (creatinine >97 µmol/L or a doubling of baseline levels), pulmonary edema, eclampsia, or persistent neurological or visual symptoms. Fetal indications included abnormal fetal testing (e.g., Category III cardiotocography), intrauterine fetal demise, or persistent reversed end-diastolic flow in the umbilical artery Doppler.

### 2.5 Statistical processing and analysis

Data processing and statistical analyses were performed using R version 4.5.2. All statistical tests were two-sided, with a significance level of 0.05 where applicable.

Missingness was assessed for each prespecified predictor before model development. Complete-case analysis was used because only one otherwise eligible participant had a missing value for one prespecified predictor, serum creatinine; all other prespecified predictors were complete. Multiple imputation was therefore not performed. Detailed predictor missingness before complete-case exclusion is presented in Supplementary Table S1.

Time to delivery was analyzed using a Cox proportional hazards model with hospital admission as time zero and the Efron method for tied event times. The locked primary model retained all six continuous predictors as linear terms. The proportional hazards assumption was assessed for each term and globally using scaled Schoenfeld residuals.

A restricted cubic spline with three knots at 140, 156.5, and 180 mmHg was examined only as an exploratory sensitivity analysis for the functional form of systolic blood pressure. Because the spline specification followed examination of the fitted relationship and provided negligible improvement in optimism-corrected performance, systolic blood pressure was retained as a linear term in the locked primary model.

Model performance was evaluated for all-cause delivery by day 2 and by day 7, with delivery by the stated horizon defined as the positive event. Apparent discrimination was assessed using Harrell's C-index (32) and binary time-horizon AUCs; calibration was summarized using calibration plots, calibration intercepts and slopes, the integrated calibration index (ICI/Eavg), and Brier scores. Internal validation used 1,000 bootstrap samples in which the model was refitted and evaluated in the bootstrap and original datasets; mean optimism was subtracted from the apparent estimate (33). Apparent confidence intervals were obtained from 2,000 nonparametric row-bootstrap samples conditional on the fitted model. Youden-derived cutoffs and sensitivity, specificity, PPV, and NPV were also assessed with bootstrap correction, but were considered exploratory development-cohort operating characteristics rather than validated clinical thresholds.

Exploratory decision-curve analysis was performed over threshold probabilities of 5%–80% for all-cause delivery by each horizon (24). “Treat” represented generic enhanced delivery preparedness rather than a treatment or decision to deliver; “treat all” and “treat none” therefore represented preparedness for all or no eligible women. The full model was compared with a gestational-age-only reference model using the same bootstrap procedure. Center-related sensitivity analyses included center as a fixed effect, stratification of the Cox baseline hazard by center, center-specific apparent performance, and leave-one-center-out internal-external validation. For graphical illustration only, development-cohort risk groups were defined using the 25th and 75th percentiles of the linear predictor; values equal to the lower boundary were assigned to the low group and values equal to the upper boundary remained in the intermediate group.

### 2.6 Ethics approval statement

The study was conducted in accordance with the Declaration of Helsinki. Ethical approval for the retrospective study protocol was obtained from the Institutional Review Board of Hanoi Obstetrics and Gynecology Hospital (Approval No. CS/PSHN/DD/26/02; March 24, 2026). At Bach Mai Hospital and Bac Ninh Obstetrics and Pediatrics Hospital, the study was reviewed through the respective institutional scientific-research and administrative governance processes, and authorization was granted for site participation and access to relevant medical records based on the study decision issued through Hanoi Medical University. No separate local ethics approval number was issued at these two participating sites. The requirement for individual informed consent was waived by the approving ethics committee because the study involved retrospective analysis of routinely collected, de-identified clinical data. Data were de-identified at each participating site before transfer and pooling for analysis.

## 3 Results

### 3.1 Clinical and laboratory characteristics of the study population

Among 267 otherwise eligible participants, one participant had a missing baseline serum creatinine value (0.37%) and was excluded before model fitting. No missing values were identified for the other prespecified predictors, resulting in a final complete-case cohort of 266 participants.

All 266 participants experienced delivery during follow-up, corresponding to an event rate of 100%, with no censoring. Eighty-four women (31.6%) delivered by day 2, a further 84 (31.6%) remained undelivered after day 2 but delivered by day 7, and 98 (36.8%) remained undelivered beyond day 7. Clinical and laboratory characteristics differed across centers (Table 1); median latency ranged from 1 day in Center 2 to 6 days in Center 3, and corticosteroid administration ranged from 52.4% to 88.5% among women with documented data. Detailed variable-level availability and the centers contributing to each Table 1 comparison are reported in Supplementary Table S2.

**Table 1:** Baseline clinical and laboratory characteristics and pregnancy outcomes of the study population.

Variable	Total cohort	Center 1	Center 2	Center 3	*p*-value
	(*n* = 266)	(*n* = 88)	(*n* = 21)	(*n* = 157)	
Baseline characteristics					
Maternal age (years)	32 (28–37)	33 (27–37)	28 (23–34)	32 (29–36)	0.075
Gestational age at admission (weeks)	31.00 (28.71–32.86)	30.50 (28.00–32.25)	32.00 (31.00–33.57)	31.00 (28.57–32.86)	0.010
First admission systolic blood pressure (mmHg)	156 (145–170)	156 (150–168)	170 (155–190)	150 (140–170)	0.009
First admission diastolic blood pressure (mmHg)	100 (90–102)	100 (95–110)	100 (100–110)	100 (90–100)	<0.001
Laboratory results					
Proteinuria (g/L)	3.00 (1.00–7.18)	3.00 (1.00–10.00)	1.00 (1.00–5.00)	2.76 (1.35–7.60)	0.291
AST (U/L)	26.0 (21.0–37.2)	27.0 (20.0–50.5)	24.0 (20.0–30.0)	26.0 (21.4–32.3)	0.411
ALT (U/L)	18.6 (11.6–33.0)	25.5 (12.0–49.8)	NA	17.9 (11.5–26.7)	0.007
Creatinine (µmol/L)	71.2 (60.0–85.0)	65.0 (55.0–86.5)	82.0 (75.0–94.0)	71.2 (62.7–82.7)	0.007
Urea (mmol/L)	5.45 (4.20–7.10)	NA	5.20 (4.00–6.30)	5.50 (4.25–7.10)	0.204
Platelet count (×10^9^/L)	204 (160–255)	202 (145–278)	164 (129–219)	207 (172–249)	0.105
Pregnancy and neonatal outcomes					
Gestational age at delivery (weeks)	32.00 (30.00–33.43)	32.00 (30.00–33.00)	33.29 (31.86–34.00)	32.00 (29.86–33.71)	0.010
Latency period (days)	5 (2–11)	4 (2–9)	1 (0–5)	6 (2–11)	0.004
Corticosteroid administration	210/253 (83.0%)	60/75 (80.0%)	11/21 (52.4%)	139/157 (88.5%)	<0.001
Birth weight (grams)	1,400 (1,000–1,700)	1,400 (1,100–1,800)	NA	1,350 (1,000–1,700)	0.115
5-minute Apgar score <7	48/263 (18.3%)	12/85 (14.1%)	5/21 (23.8%)	31/157 (19.7%)	0.417
Neonatal death	17/266 (6.4%)	9/88 (10.2%)	1/21 (4.8%)	7/157 (4.5%)	0.183

Data are presented as median (interquartile range, IQR) for continuous variables and n/N (%) for categorical variables. Denominators vary according to data availability, and missing values were excluded from percentage calculations. Group comparisons were performed using the Kruskal–Wallis test for continuous variables and the chi-square test or Fisher's exact test for categorical variables, as appropriate. *p* values were calculated using available-case data. Because some variables were unavailable at selected centers, ALT and birth weight were compared between Centers 1 and 3, whereas urea was compared between Centers 2 and 3. Birth weight was measured after delivery. Neonatal death was defined as death occurring within 24 h after birth. AST, aspartate aminotransferase; ALT, alanine aminotransferase; NA, not available.

### 3.2 Predictor analysis and model development

In the primary model, systolic blood pressure was modeled linearly. In the exploratory spline sensitivity analysis, the overall systolic blood pressure association was significant (*p* = 0.0013), whereas the nonlinear component was not (*p* = 0.077). The spline increased the optimism-corrected AUC by only 0.004 at day 2 and <0.001 at day 7 compared with the linear specification (Supplementary Figure S1 and Supplementary Table S3).

In the primary multivariable Cox model, gestational age at admission contributed most strongly (*χ*^2^ = 47.54; p < 0.0001), followed by proteinuria (*χ*^2^ = 23.78), AST (*χ*^2^ = 16.67), platelet count (*χ*^2^ = 15.76), systolic blood pressure (*χ*^2^ = 8.72), and creatinine (*χ*^2^ = 1.33) (Supplementary Table S4).

In the multivariable Cox model (Table 2), the adjusted hazard ratio comparing the 75th with the 25th percentile was 2.20 (95% CI 1.76–2.76) for gestational age, 1.30 (95% CI 1.09–1.54) for systolic blood pressure, 1.36 (95% CI 1.20–1.54) for proteinuria, 1.09 (95% CI 1.05–1.14) for AST, 1.02 (95% CI 0.99–1.05) for creatinine, and 0.71 (95% CI 0.60–0.84) for platelet count. No proportional-hazards violation was detected (global *χ*^2^ = 3.69, df = 6, *p* = 0.719; Supplementary Table S5).

**Table 2:** Adjusted hazard ratios for delivery in the multivariable Cox regression model.

Predictor	Comparison (Q3 vs. Q1)	Adjusted HR (95% CI)	*p*-value
Systolic blood pressure (mmHg)	170.00 versus 145.00	1.30 (1.09–1.54)	0.003
Platelet count (G/L)	254.75 versus 160.00	0.71 (0.60–0.84)	<0.0001
Proteinuria (g/L)	7.18 versus 1.00	1.36 (1.20–1.54)	<0.0001
AST (U/L)	37.15 versus 21.02	1.09 (1.05–1.14)	<0.0001
Creatinine (µmol/L)	85.00 versus 60.00	1.02 (0.99–1.05)	0.248
Gestational age at admission (weeks)	32.86 versus 28.71	2.20 (1.76–2.76)	<0.0001

Hazard ratios compare the 75th with the 25th percentile of each continuous predictor in the locked primary linear-SBP Cox model. *AST*, aspartate aminotransferase.

### 3.3 The LATE-PE predictive tool

**Figure 2:**
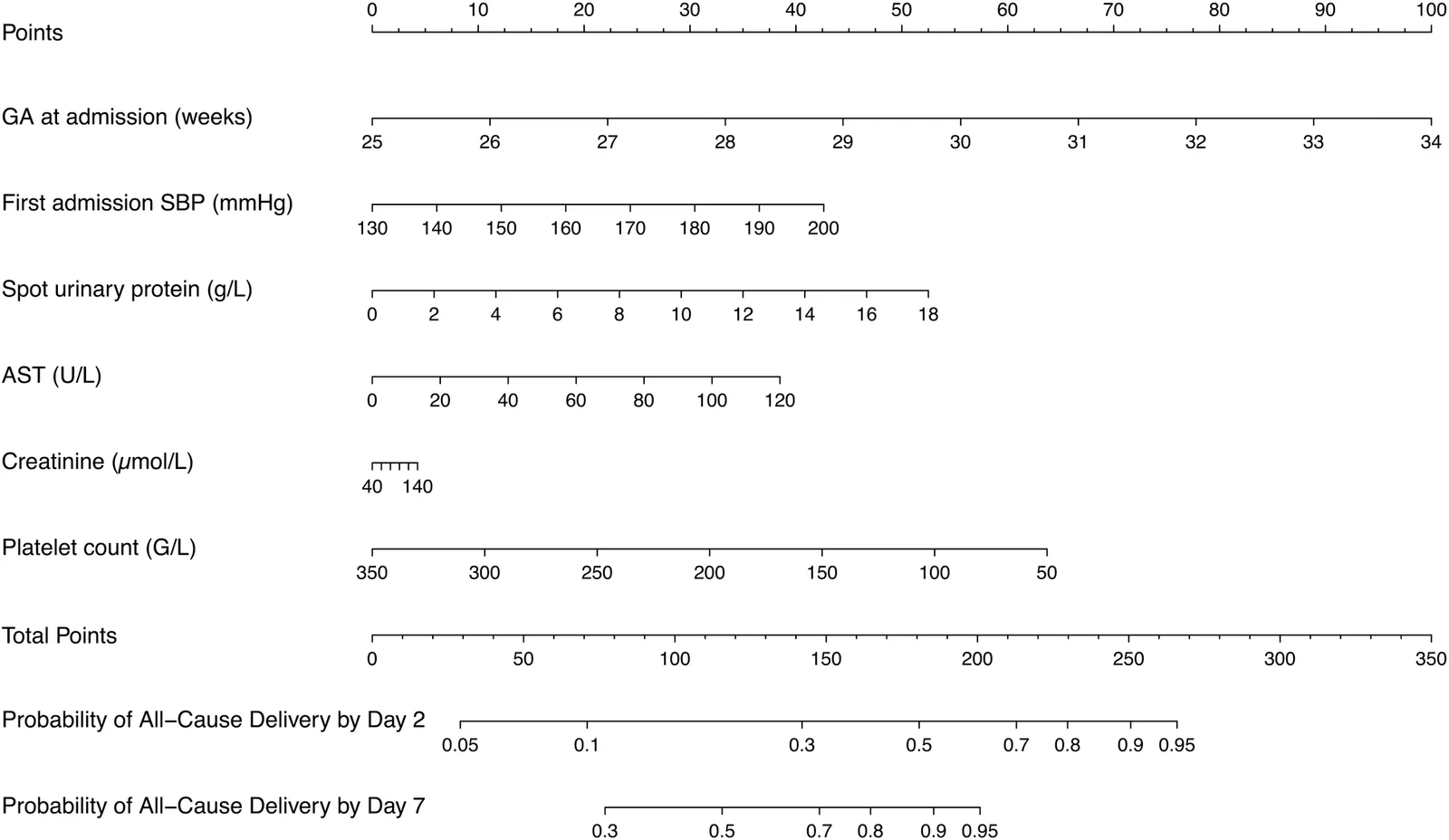
Nomogram for predicting the probability of all-cause delivery by day 2 and by day 7 after admission (LATE-PE model). The displayed predictor ranges were based on the observed 5th–95th percentiles, with rounded axis limits for readability. GA, gestational age at admission; SBP, systolic blood pressure.

The LATE-PE model was presented as a nomogram for the predicted probability of all-cause delivery by day 2 and by day 7 (Figure 2). The predictor axes display values from the observed 5th to 95th percentiles, while the complete observed ranges are reported in Supplementary Table S6. The full mathematical equations for calculating the linear predictor and the individual predicted probabilities are provided in the Supplementary Table S7 and Supplementary Text.

### 3.4 Internal validation and model performance

**Figure 3:**
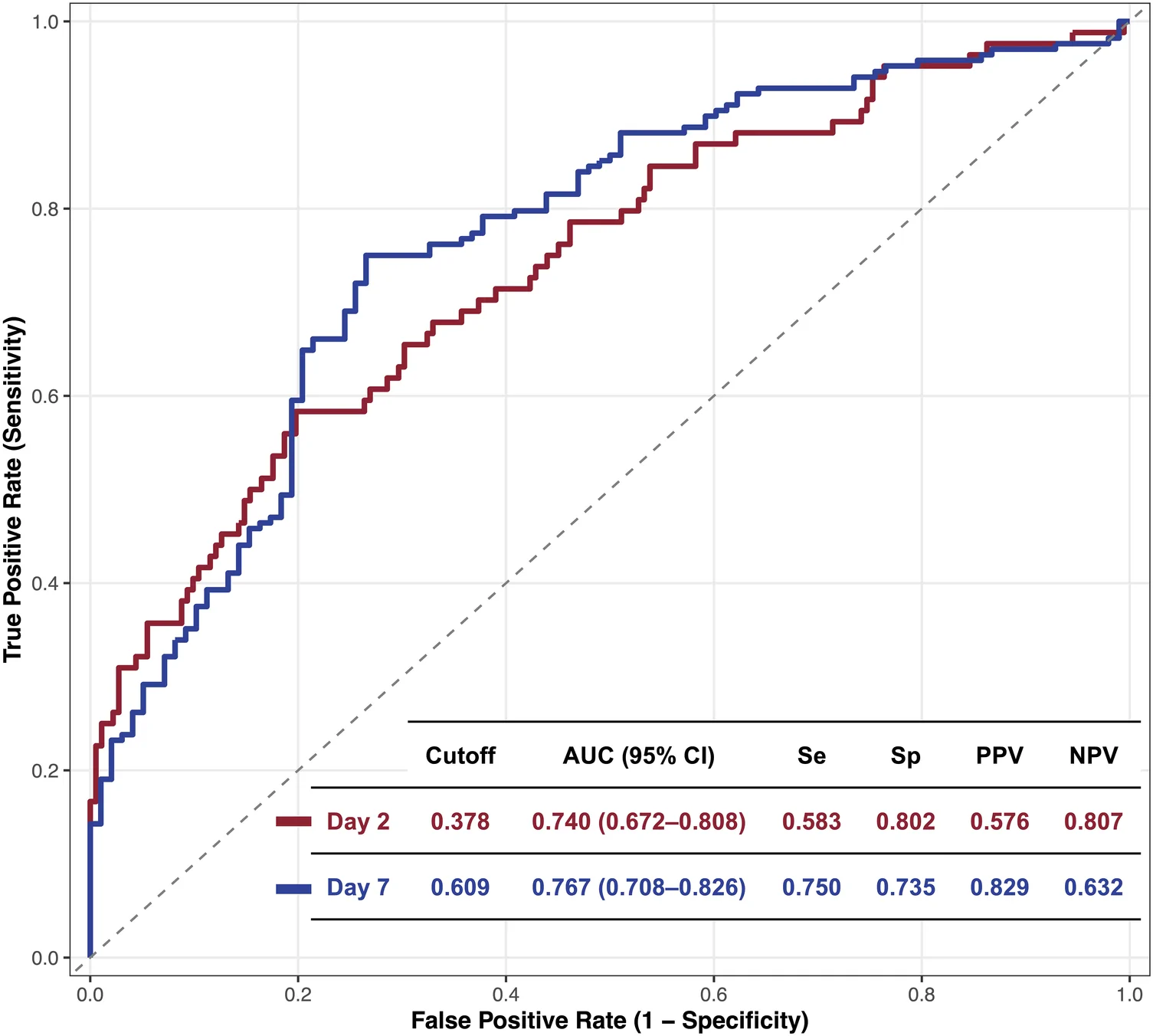
Apparent binary ROC curves for all-cause delivery by day 2 and by day 7 after admission. The plotted curves and AUCs use the same binary horizon definitions. AUC, area under the receiver operating characteristic curve; Se, sensitivity; Sp, specificity; PPV, positive predictive value; NPV, negative predictive value.

Model performance is summarized in Tables 3, 4. The apparent Harrell's C-index was 0.695 (95% CI 0.655–0.736), with an optimism-corrected value of 0.689. For all-cause delivery by day 2, the apparent AUC was 0.740 (95% CI 0.672–0.808), and the optimism-corrected AUC was 0.732. At day 7, the corresponding AUC estimates were 0.767 (95% CI 0.708–0.826) and 0.758, respectively (Figure 3).

**Table 3:** Apparent and bootstrap optimism-corrected performance of the LATE-PE model.

Performance measure	Apparent (95% CI)	Optimism-corrected
Harrell's C-index (overall follow-up)	0.695 (0.655–0.736)	0.689
AUC, day 2	0.740 (0.672–0.808)	0.732
AUC, day 7	0.767 (0.708–0.826)	0.758
Calibration intercept, day 2	−0.051 (−0.344–0.221)	−0.058
Calibration intercept, day 7	−0.015 (−0.299–0.288)	−0.023
Calibration slope, day 2	1.125 (0.807–1.555)	1.023
Calibration slope, day 7	0.938 (0.668–1.338)	0.806
Brier score, day 2	0.174 (0.151–0.199)	0.180
Brier score, day 7	0.187 (0.164–0.208)	0.193

Values are presented as apparent estimates with 95% confidence intervals and bootstrap optimism-corrected estimates. AUC, area under the receiver operating characteristic curve.

**Table 4:** Exploratory operating characteristics of the LATE-PE model at the Youden-derived cutoffs.

Horizon	Estimate	Sensitivity	Specificity	NPV	PPV
Day 2, cutoff 0.378	Apparent (95% CI)	0.583 (0.474–0.684)	0.802 (0.745–0.854)	0.807 (0.750–0.862)	0.576 (0.469–0.675)
	Optimism-corrected	0.548	0.786	0.790	0.544
Day 7, cutoff 0.609	Apparent (95% CI)	0.750 (0.683–0.815)	0.735 (0.648–0.816)	0.632 (0.541–0.718)	0.829 (0.767–0.885)
	Optimism-corrected	0.735	0.714	0.612	0.814

Values are presented as apparent estimates with 95% confidence intervals and bootstrap optimism-corrected estimates. Youden-derived cutoffs and their operating characteristics are exploratory. NPV, negative predictive value; PPV, positive predictive value.

The apparent calibration intercepts were −0.051 at day 2 and −0.015 at day 7, with optimism-corrected estimates of −0.058 and −0.023, respectively. The apparent calibration slopes were 1.125 at day 2 and 0.938 at day 7; after bootstrap correction, the corresponding slopes were 1.023 and 0.806. The optimism-corrected Brier scores were 0.180 at day 2 and 0.193 at day 7. Apparent ICI values were 0.039 and 0.007, respectively, and were not corrected for optimism. Apparent calibration plots are presented in Supplementary Figure S2.

At the internally selected Youden cutoff of 0.378 for delivery by day 2, the apparent sensitivity, specificity, PPV, and NPV were 0.583, 0.802, 0.576, and 0.807, respectively. The corresponding optimism-corrected estimates were 0.548, 0.786, 0.544, and 0.790. At the day-7 cutoff of 0.609, the apparent sensitivity, specificity, PPV, and NPV were 0.750, 0.735, 0.829, and 0.632, with optimism-corrected estimates of 0.735, 0.714, 0.814, and 0.612, respectively. These cutoffs and operating characteristics were derived from the development cohort and are therefore exploratory rather than validated clinical thresholds.

### 3.5 Potential clinical utility and risk stratification

**Figure 4:**
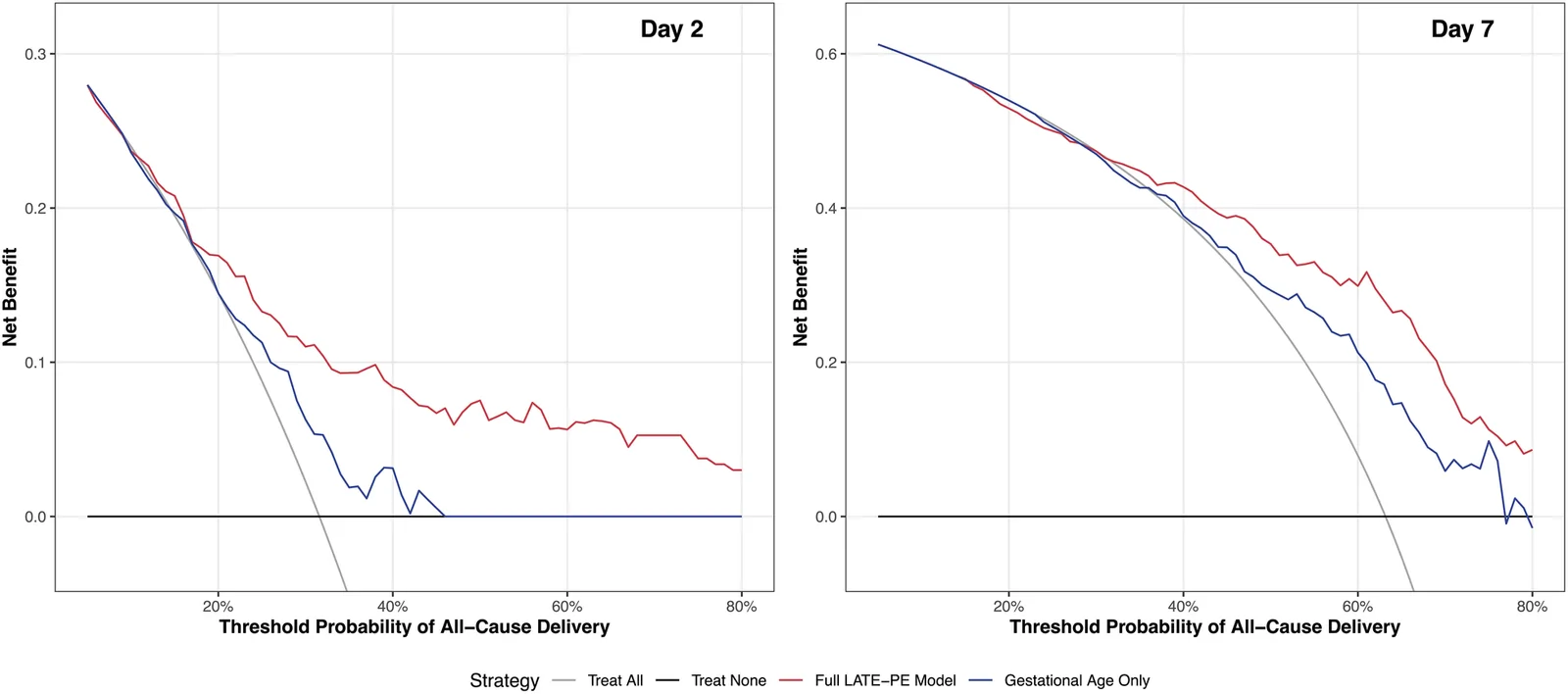
Exploratory decision-curve analysis for all-cause delivery by day 2 and by day 7. “Treat” denotes generic enhanced delivery preparedness rather than a decision to deliver.

**Figure 5:**
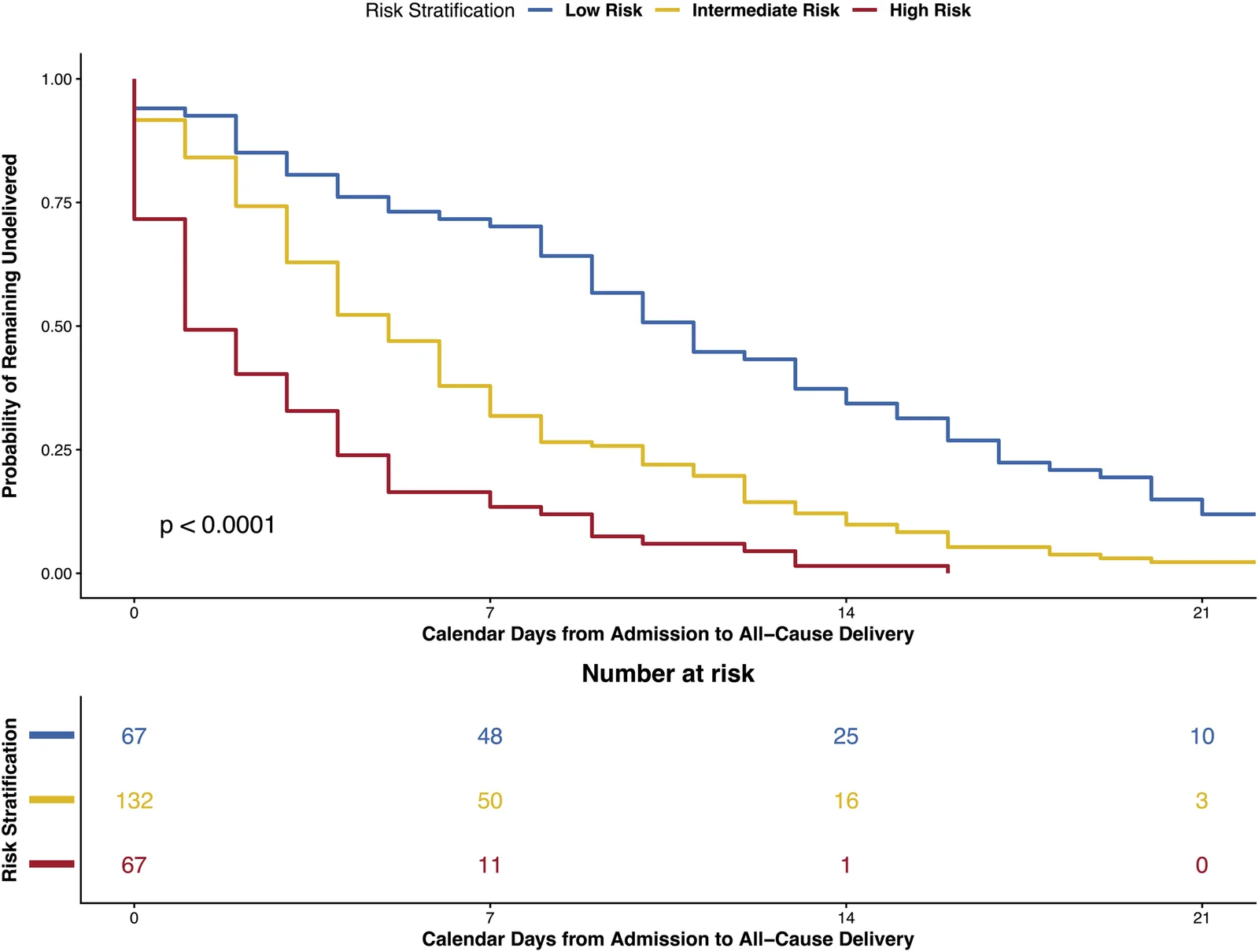
Exploratory development-cohort Kaplan–Meier curves based on the 25th and 75th percentiles of the LATE-PE linear predictor. The groups are descriptive and do not constitute independent validation.

Exploratory decision curves suggested higher net benefit for the full model than for treat-all, treat-none, and gestational-age-only strategies over portions of the evaluated threshold range (Figure 4). Compared with gestational age alone, the optimism-corrected AUC increased by 0.109 at day 2 and 0.083 at day 7 (Supplementary Table S8). Center-adjusted and center-stratified models produced similar predictor coefficients. Center-specific apparent AUCs ranged from 0.648 to 0.857 at day 2 and from 0.721 to 0.782 at day 7; leave-one-center-out AUCs ranged from 0.627 to 0.847 and from 0.711 to 0.775, respectively, with unstable estimates for Center 2 because *n* = 21 (Supplementary Table S9). Exploratory risk groups comprised 67 low-, 132 intermediate-, and 67 high-risk participants and were derived in the development cohort (Figure 5 and Supplementary Table S10).

## 4 Discussion

Our study developed and internally validated a specialized predictive model (LATE-PE) to estimate the probability of all-cause delivery by day 2 and by day 7 after admission in women with early-onset preeclampsia who had already been considered eligible for expectant management. Predicting the latency period in this population has attracted increasing attention in recent research; however, clinical feasibility remains a challenge. For instance, the model by Villalaín et al. (19) demonstrated good performance in predicting the 7-day milestone (AUC 0.79), but required a large number of variables, including Doppler ultrasound and sFlt-1/PlGF biomarkers (19). Similarly, although the study by Hackelöer et al. (25) reduced the number of input variables, its performance remained dependent on these specialized tests (25). The reliance on such biomarkers, which may be costly and not widely available, may limit their implementation in routine clinical practice, particularly in resource-constrained settings. On the other hand, efforts to eliminate the need for biomarkers, such as the PEDeliveryTime model by Yang et al. (26), which achieved a C-index of 0.76 using electronic health record data, have employed deep learning approaches with limited interpretability (“black-box” characteristics) and a relatively large number of input variables (26). In this context, LATE-PE offers a parsimonious alternative based on six routinely available clinical and laboratory variables: gestational age, systolic blood pressure, proteinuria, AST, creatinine, and platelet count. The model achieved apparent AUCs of 0.740 at day 2 and 0.767 at day 7, with corresponding optimism-corrected values of 0.732 and 0.758. Its reliance on routinely collected variables may facilitate implementation across healthcare settings with differing levels of resources. Nevertheless, these estimates reflect performance within the development cohort and do not yet establish clinical effectiveness or transportability. Overall, LATE-PE demonstrated moderate discrimination and warrants further validation in independent populations.

An important strength of the LATE-PE model lies in its variable selection and processing strategy, which is grounded in pathophysiological rationale rather than data-driven selection. Guided by international recommendations and prior literature (7, 20), the six predictors were selected *a priori* based on routine availability and their capacity to reflect gestational age, blood-pressure severity, proteinuria, hepatic and renal dysfunction, and thrombocytopenia. Specifically, serum creatinine was retained in the model despite not reaching statistical significance in multivariable analysis. As a key biomarker reflecting renal dysfunction, excluding creatinine solely based on statistical significance may reduce the clinical interpretability of the model and is generally discouraged in contemporary prediction modeling frameworks (27, 28). Multivariable analysis identified gestational age at admission, platelet count, proteinuria, and AST as important prognostic factors for the latency period, consistent with previous studies (29, 30). Systolic blood pressure was modeled linearly in the primary model. An exploratory restricted cubic spline analysis showed no clear evidence of nonlinearity and produced negligible improvement in optimism-corrected performance; consequently, the fitted spline pattern should not be interpreted as establishing a validated clinical blood-pressure threshold.

The real-world management of early-onset preeclampsia remains a significant challenge, requiring a balance between maternal risks and fetal benefits, and often leading to variability in clinical decision-making. The participating centers differed in latency duration, corticosteroid administration, systolic blood pressure, and serum creatinine levels. These differences may reflect variation in case mix, referral pathways, institutional protocols, resource availability, and clinician-mediated decisions regarding delivery. Because time to delivery is partly determined by management practice, LATE-PE estimates prognosis under the clinical settings represented in this cohort rather than the untreated natural history of early-onset preeclampsia. Predictor coefficients were broadly similar in center-adjusted and center-stratified sensitivity analyses; however, center-specific and leave-one-center-out performance estimates varied and were imprecise for Center 2 because of its small sample. Therefore, the acceptable pooled internal performance observed in this study does not establish transportability across centers or healthcare systems.

At the day-2 milestone, the internally selected cutoff yielded an apparent negative predictive value of 0.807, with an optimism-corrected estimate of 0.790, indicating that women classified as lower risk were relatively likely to remain undelivered beyond day 2. This may be relevant when considering whether there is sufficient time to complete antenatal corticosteroid therapy, although it should not be used to justify expectant management in the presence of maternal or fetal indications for delivery. Conversely, at the 7-day milestone, the apparent positive predictive value was 0.829, with an optimism-corrected estimate of 0.814, indicating a relatively high probability of delivery within one week among women classified as high risk. These estimates may help inform planning for antenatal corticosteroid completion, magnesium sulfate administration, and *in utero* transfer, but should be interpreted alongside comprehensive maternal and fetal assessment. Because the cutoffs and operating characteristics were derived from the development cohort, they remain exploratory and should not be regarded as validated clinical thresholds.

Decision curve analysis suggested that LATE-PE may provide a higher net benefit than “treat-all,” “treat-none,” and gestational-age-only strategies across selected portions of the evaluated threshold range (24). For this exploratory analysis, a model-positive result was interpreted as prompting enhanced preparedness for delivery rather than recommending delivery or a specific treatment; “treat-all” and “treat-none” represented applying this preparedness strategy to all or none of the eligible women, respectively. However, decision curve analysis evaluates model-based strategies under specified assumptions and does not by itself establish improved maternal or neonatal outcomes. Decisions regarding expectant management and delivery in early-onset preeclampsia remain multifactorial and should not rely solely on model predictions; they require integration of maternal symptoms, blood pressure control, serial laboratory trends, fetal status, gestational age, institutional resources, and clinician judgment. A recent study by Karakaya and Katirci evaluated the Naples Prognostic Score in Obstetrics, which combines routinely available inflammatory and nutritional parameters to predict subsequent preeclampsia development (31). Although its target outcome differs from that of LATE-PE, both approaches illustrate the potential value of pragmatic prediction tools based on accessible clinical data and the need for validation before routine implementation. Accordingly, the LATE-PE web application (https://chienchu.shinyapps.io/LATE_PE_WebApp/) remains an experimental research-use prototype intended to facilitate model exploration and future external validation. It should not be used as a stand-alone clinical decision-support tool or override established indications for urgent delivery.

Our study has several strengths. First, the use of multicenter data from three medical facilities captured variation in case mix and practice patterns. Second, the model used prespecified, routinely available predictors and retained continuous information, while potential nonlinearity in systolic blood pressure was examined in an exploratory restricted cubic spline sensitivity analysis. Third, the complete model specification was reported, and internal validation using 1,000 bootstrap resamples allowed estimation of optimism and assessment of model stability. However, several limitations should be acknowledged. Time to delivery is a clinician-mediated outcome influenced by maternal and fetal status, clinician judgment, and institutional protocols. The observed between-center differences in latency, corticosteroid use, and baseline characteristics, together with the relatively small sample from Center 2, may have affected model estimates. The model was developed using pooled multicenter data, therefore residual heterogeneity in clinical practice may persist. Latency was available only at day-level resolution, and day 2 was consequently used as an operational approximation of the clinically relevant 48-hour window. The outcome was defined as all-cause delivery because maternal, fetal, spontaneous, and combined indications could not be classified consistently across centers. Laboratory analyzers were not identical across hospitals, although measurement units were harmonized and national internal and external quality-control requirements were followed. The retrospective design also permits residual measurement error, unmeasured confounding, and incomplete capture of management-related factors. Finally, the Youden-derived cutoffs, decision curves, and percentile-based risk groups were generated within the development cohort and remain exploratory. LATE-PE has undergone internal validation only; its discrimination, calibration, transportability, and clinical impact should be evaluated in independent populations before routine clinical implementation.

## 5 Conclusion

This study developed and internally validated LATE-PE for estimating all-cause delivery by day 2 and by day 7 after admission in women with early-onset preeclampsia already considered eligible for expectant management. The model uses six routinely available predictors and showed optimism-corrected AUCs of 0.732 and 0.758. The web application should be regarded as an experimental research-use prototype, and external validation in independent populations and prospective assessment of clinical impact are required before the model can be used to support patient management.

## Data Availability

The raw data supporting the conclusions of this article will be made available by the authors, without undue reservation.
