# Supplementary material for: Development and internal validation of the LATE-PE model for predicting time-to-delivery in early-onset preeclampsia: a multicenter study
Source: Front Med (Lausanne). 2026 Aug 28;13:1870604. doi: 10.3389/fmed.2026.1870604 (PMC13562031; doi:10.3389/fmed.2026.1870604)
Supplement: Supplementary file 1 [file Datasheet1.docx]

Supplementary Material

# 1 Supplementary Data

The datasets presented in this article are not readily available due to privacy and ethical restrictions regarding patient data. Requests to access the datasets should be directed to the corresponding authors.

# 2 Supplementary Figures and Tables

## 2.1 Supplementary Figures

**Supplementary Figure 1.** Exploratory restricted-cubic-spline sensitivity analysis for first admission systolic blood pressure. The curve displays adjusted hazard ratios for all-cause delivery relative to 140 mmHg with 95% confidence intervals. This exploratory curve does not define a validated clinical blood-pressure cutoff.

**Supplementary Figure 2.** Apparent calibration plots for all-cause delivery by day 2 and by day 7 after admission. The red dashed line represents ideal calibration, the solid black line represents the fitted apparent calibration curve, and the grey shaded area represents the 95% confidence interval. Optimism-corrected calibration slopes are reported in **Table 3** of the main manuscript.

## 2.2 Supplementary Tables

## Supplementary Table 1. Missingness of prespecified predictors before complete-case exclusion.

| **Prespecified predictor** | **Otherwise eligible, n** | **Observed, n** | **Missing, n** | **Missing, %** |
| --- | --- | --- | --- | --- |
| GA (weeks) | 267 | 267 | 0 | 0.00 |
| SBP (mmHg) | 267 | 267 | 0 | 0.00 |
| Proteinuria (g/L) | 267 | 267 | 0 | 0.00 |
| AST (U/L) | 267 | 267 | 0 | 0.00 |
| Creatinine (µmol/L) | 267 | 266 | 1 | 0.37 |
| Platelet count (G/L) | 267 | 267 | 0 | 0.00 |
| *GA*, gestational age; *SBP*, systolic blood pressure; *AST*, aspartate aminotransferase. | | | | |

## Supplementary Table 2. Variable-level missingness and centers contributing to Table 1 summaries and comparisons

| Variable | Overall missing | Center 1 missing | Center 2 missing | Center 3 missing | Centers contributing to comparison |
| --- | --- | --- | --- | --- | --- |
| Baseline characteristics | | | | | |
| Maternal age (years) | 0/266 | 0/88 | 0/21 | 0/157 | Centers 1, 2, and 3 |
| Gestational age at admission (weeks) | 0/266 | 0/88 | 0/21 | 0/157 | Centers 1, 2, and 3 |
| First admission systolic blood pressure (mmHg) | 0/266 | 0/88 | 0/21 | 0/157 | Centers 1, 2, and 3 |
| First admission diastolic blood pressure (mmHg) | 0/266 | 0/88 | 0/21 | 0/157 | Centers 1, 2, and 3 |
| Laboratory results | | | | | |
| Spot urinary protein concentration (g/L) | 0/266 | 0/88 | 0/21 | 0/157 | Centers 1, 2, and 3 |
| AST (U/L) | 0/266 | 0/88 | 0/21 | 0/157 | Centers 1, 2, and 3 |
| ALT (U/L) | 21/266 | 0/88 | 21/21 | 0/157 | Centers 1 and 3 |
| Creatinine (µmol/L) | 0/266 | 0/88 | 0/21 | 0/157 | Centers 1, 2, and 3 |
| Urea (mmol/L) | 90/266 | 88/88 | 0/21 | 2/157 | Centers 2 and 3 |
| Platelet count (×10^9/L) | 0/266 | 0/88 | 0/21 | 0/157 | Centers 1, 2, and 3 |
| Pregnancy and neonatal outcomes | | | | | |
| Gestational age at delivery (weeks) | 0/266 | 0/88 | 0/21 | 0/157 | Centers 1, 2, and 3 |
| Admission-to-delivery latency (calendar days) | 0/266 | 0/88 | 0/21 | 0/157 | Centers 1, 2, and 3 |
| Antenatal corticosteroid administration | 13/266 | 13/88 | 0/21 | 0/157 | Centers 1, 2, and 3 |
| Birth weight measured after delivery (g) | 21/266 | 0/88 | 21/21 | 0/157 | Centers 1 and 3 |
| 5-minute Apgar score <7 | 3/266 | 3/88 | 0/21 | 0/157 | Centers 1, 2, and 3 |
| Neonatal death within 24 hours after birth | 0/266 | 0/88 | 0/21 | 0/157 | Centers 1, 2, and 3 |
| Values are missing n/N. Comparisons used available-case data. ALT and birth weight were compared between Centers 1 and 3, whereas urea was compared between Centers 2 and 3. Birth weight was measured after delivery. Neonatal death was defined as death occurring within 24 hours after birth. *AST*, aspartate aminotransferase; *ALT*, alanine aminotransferase. | | | | | |

## Supplementary Table 3. Exploratory restricted-cubic-spline sensitivity analysis for systolic blood pressure.

**Panel A.** Spline association tests and hazard-ratio contrast

| **Comparison** | **Adjusted HR (95% CI)** | **Overall SBP p-value** | **Nonlinearity p-value** |
| --- | --- | --- | --- |
| 170.00 versus 145.00 mmHg | 1.19 (0.98–1.44) | 0.0013 | 0.077 |
| The HR compares the 75th with the 25th percentile. The overall p-value tests the complete spline term; the nonlinearity p-value tests only the nonlinear component. | | | |

**Panel B.** Bootstrap optimism-corrected performance of the linear and spline specifications

| **Metric** | **Primary linear SBP** | **Spline sensitivity** | **Spline minus linear** |
| --- | --- | --- | --- |
| C-index | 0.6889 | 0.6908 | +0.0020 |
| AUC, day 2 | 0.7317 | 0.7360 | +0.0043 |
| AUC, day 7 | 0.7584 | 0.7587 | +0.0003 |
| Brier score, day 2 | 0.1800 | 0.1801 | +0.0001 |
| Brier score, day 7 | 0.1926 | 0.1929 | +0.0003 |
| The locked primary model retained linear systolic blood pressure because the exploratory spline showed no clear evidence of nonlinearity and negligible improvement in optimism-corrected performance. | | | |

## Supplementary Table 4. Wald Analysis of Variance (ANOVA) for predictor variables in the final multivariable Cox regression model

| **Predictor** | **χ^2^** | **df** | **p-value** |
| --- | --- | --- | --- |
| GA (weeks) | 47.54 | 1 | <0.0001 |
| SBP (mmHg) | 8.72 | 1 | 0.0031 |
| Proteinuria (g/L) | 23.78 | 1 | <0.0001 |
| AST (U/L) | 16.67 | 1 | <0.0001 |
| Creatinine (µmol/L) | 1.33 | 1 | 0.2484 |
| Platelet count (G/L) | 15.76 | 1 | 0.0001 |
| Total | 100.14 | 6 | <0.0001 |
| *GA*, Gestational Age; *SBP*, Systolic Blood Pressure; *AST*, Aspartate Aminotransferase | | | |

## Supplementary Table 5. Assessment of the proportional hazards assumption for the locked primary model using scaled Schoenfeld residuals

| **Predictor** | **χ^2^** | **df** | **p-value** |
| --- | --- | --- | --- |
| GA at admission (weeks) | 0.576 | 1 | 0.448 |
| SBP (mmHg), linear | 2.232 | 1 | 0.135 |
| Proteinuria (g/L) | 0.175 | 1 | 0.676 |
| AST (U/L) | 0.112 | 1 | 0.738 |
| Creatinine (µmol/L) | 0.074 | 1 | 0.786 |
| Platelet count (G/L) | 0.337 | 1 | 0.562 |
| Global test | 3.689 | 6 | 0.719 |
| *GA*, Gestational Age; *SBP*, Systolic Blood Pressure; *AST*, Aspartate Aminotransferase | | | |

## Supplementary Table 6. Observed predictor ranges in the model-development cohort

| **Predictor** | **Min** | **5th percentile** | **Median** | **95th percentile** | **Max** |
| --- | --- | --- | --- | --- | --- |
| GA (weeks) | 22.00 | 25.71 | 31.00 | 33.86 | 34.00 |
| First admission SBP (mmHg) | 110.00 | 136.25 | 156.50 | 200.00 | 224.00 |
| Spot urinary protein (g/L) | 0.00 | 0.30 | 3.00 | 16.18 | 31.60 |
| AST (U/L) | 6.60 | 16.00 | 26.00 | 115.00 | 400.00 |
| Creatinine (µmol/L) | 30.00 | 46.00 | 71.25 | 135.25 | 938.00 |
| Platelet count (×10^9^/L) | 35.00 | 78.00 | 204.50 | 334.75 | 551.00 |
| The main nomogram displays the observed 5th–95th percentile range. Predictions near the minimum and maximum values represent sparse distributional extremes and should be interpreted cautiously. *GA*, Gestational Age; *SBP*, Systolic Blood Pressure; *AST*, Aspartate Aminotransferase | | | | | |

## Supplementary Table 7. Complete calculation specification of the locked primary LATE-PE model

| **Component** | **Estimate** |
| --- | --- |
| LP implementation constant | −7.27701322631838 |
| Coefficient: GA at admission | 0.190805443055117 |
| Coefficient: SBP | 0.0104577122504363 |
| Coefficient: spot urinary protein concentration | 0.050092441434894 |
| Coefficient: AST | 0.00550794056213781 |
| Coefficient: serum creatinine | 0.000734088509425144 |
| Coefficient: platelet count | −0.00364686545369123 |
| Baseline survival at day 2 for LP=0 | 0.710952512359764 |
| Baseline survival at day 7 for LP=0 | 0.352221061842671 |
| The LP constant is the implementation centering term used to reproduce predictions from the fitted model; a Cox model does not contain a conventional intercept. *LP*, Linear Predictor; *GA*, Gestational Age; *SBP*, Systolic Blood Pressure; *AST*, Aspartate Aminotransferase | |

## Supplementary Table 8. Incremental predictive value of the locked primary model beyond gestational age alone

| **Metric** | **GA-only corrected** | **Full model corrected** | **Corrected difference** | **Paired bootstrap 95% CI*** |
| --- | --- | --- | --- | --- |
| AUC, day 2 | 0.623 | 0.732 | 0.109 | 0.050 to 0.186 |
| AUC, day 7 | 0.675 | 0.758 | 0.083 | 0.037 to 0.151 |
| Brier score, day 2 | 0.210 | 0.180 | −0.030 | −0.050 to −0.018 |
| Brier score, day 7 | 0.215 | 0.193 | −0.023 | −0.041 to −0.009 |
| C-index | 0.626 | 0.689 | 0.063 | 0.032 to 0.108 |
| *The confidence interval is for the apparent paired difference from 2,000 nonparametric row-bootstrap samples, conditional on the two fitted models. Positive differences favor the full model for AUC and C-index; negative differences favor the full model for Brier score. *GA*, Gestational Age. | | | | |

## Supplementary Table 9. Exploratory center-sensitivity and leave-one-center-out analyses

**Panel A.** Predictor coefficients across pooled and center-sensitive Cox models

| **Predictor** | **Pooled model β** | **Center fixed-effect β** | **Center-stratified β** |
| --- | --- | --- | --- |
| Gestational age at admission | 0.190805 | 0.197858 | 0.211191 |
| Systolic blood pressure | 0.010458 | 0.010580 | 0.010415 |
| Spot urinary protein concentration | 0.050092 | 0.050582 | 0.051814 |
| AST | 0.005508 | 0.005305 | 0.004081 |
| Creatinine | 0.000734 | 0.000586 | 0.000692 |
| Platelet count | −0.003647 | −0.003687 | −0.003801 |

**Panel B.** Center-specific apparent discrimination and leave-one-center-out discrimination

| **Center (n)** | **Apparent AUC day 2 (95% CI)** | **Apparent AUC day 7 (95% CI)** | **LOCO AUC day 2** | **LOCO AUC day 7** |
| --- | --- | --- | --- | --- |
| Center 1 (n=88) | 0.648 (0.511–0.782) | 0.730 (0.612–0.830) | 0.627 | 0.711 |
| Center 2 (n=21) | 0.857 (0.667–1.000) | 0.721 (0.421–1.000) | 0.847 | 0.721 |
| Center 3 (n=157) | 0.765 (0.671–0.847) | 0.782 (0.705–0.854) | 0.755 | 0.775 |
| Center 2 estimates are imprecise because only 21 participants were available. LOCO estimates were not additionally corrected for optimism within each held-out analysis. *LOCO*, leave-one-center-out internal-external validation. | | | | |

## Supplementary Table 10. Exploratory development-cohort risk-group definitions and counts

| **Exploratory group** | **Linear-predictor rule** | **n** | **%** |
| --- | --- | --- | --- |
| Low risk | LP ≤ −0.5163 | 67 | 25.2 |
| Intermediate risk | −0.5163 < LP ≤ 0.4655 | 132 | 49.6 |
| High risk | LP > 0.4655 | 67 | 25.2 |
| Groups were defined from the same cohort used to develop the model and do not constitute independent validation. Equality at the lower boundary was assigned to low risk; equality at the upper boundary remained intermediate. *LP*, Linear Predictor | | | |

## 3. Supplementary Text

**Mathematical Formulation of the LATE-PE Risk Prediction Model**

The locked primary LATE-PE model estimates the probability of all-cause delivery by day 2 and by day 7 after hospital admission among women with early-onset preeclampsia who had already been considered eligible for expectant management. Day 2 is an operational calendar-day approximation of the clinically relevant 48-hour window because exact admission and delivery times were unavailable. The model uses a Cox proportional hazards framework with six linear continuous predictors.

**Step 1: Calculation of the Linear Predictor**

Predictor values should be entered in the units shown below. Systolic blood pressure is linear in the locked primary model. The full numerical specification is also provided in **Supplementary Table 7**.

Based on the model fitting, the exact formula for calculating the LP is as follows:

$$LP = -7.2770132263 + 0.1908054431\times GA + 0.0104577123\times SBP + 0.0500924414\times Proteinuria + 0.0055079406\times AST + 0.0007340885\times Creatinine - 0.0036468655\times Platelet$$

(*Note: GA is gestational age at admission in weeks; SBP is the first admission systolic blood pressure in mmHg before antihypertensive treatment administered at the study hospital; Proteinuria is spot urinary protein concentration in g/L; AST is in U/L; Creatinine is in µmol/L; and Platelet is in ×10^9/L.)*

**Step 2: Calculation of the Predicted Probability**

Once the LP is determined for a specific patient, the probability of delivery within t days (Pₜ) can be calculated using the baseline survival function S₀(t) according to the standard Cox model transformation:

$$P(Delivery \leq t)=1- S_{0}(t)^{exp(LP)}$$

Based on our final model, the baseline survival probabilities for the two critical clinical thresholds are:

- Baseline survival at day 2 (t = 2 days): S_0_ (2) = 0.7109525124
- Baseline survival at day 7 (t = 7 days): S_0_ (7) = 0.3522210618

Thus, the exact probabilities can be computed manually or programmed into a clinical decision support system (such as web or mobile applications) using the following equations:

Probability of delivery within 2 days:

$$P_{2 days}=1- {0.7109525124}^{exp(LP)}$$

Probability of delivery within 7 days:

$$P_{7 days}=1- {0.3522210618}^{exp(LP)}$$

These equations reproduce the model and are intended for research, model exploration, and external validation. They should not be used as a stand-alone basis for delivery decisions or to override established maternal or fetal indications for urgent delivery.
